# Evaluation of Physical Properties of Denture Base Resins Containing Silver Nanoparticles of Aloe barbadensis Miller, Morinda citrifolia, and Boesenbergia rotunda and Its Anti-microbial Effect: An In Vitro Study

**DOI:** 10.7759/cureus.48260

**Published:** 2023-11-04

**Authors:** Saguna Kaul, Shafath Ahmed, Vidyashree V Nandini, Jailance Lathief, Shiney Boruah

**Affiliations:** 1 Prosthodontics, Sri Ramaswamy Memorial (SRM) Kattankulathur Dental College, Chennai, IND

**Keywords:** polymethylmethacrylate (pmma), finger root, noni, aloe vera, silver nanoparticles, hardness, candida albicans, antifungal, flexural strength

## Abstract

Introduction

The denture bases fabricated from polymethylmethacrylate (PMMA) have some disadvantages, such as surface prone to microbial growth and biofilm accumulation, which contributes to the onset and dissemination of infections among denture wearers. Therefore, the purpose of this in vitro study was to evaluate the flexural strength, hardness, and antimicrobial effect of denture base resin incorporated with 0.05% and 0.1% silver nanoparticles (AgNPs) of Aloe barbadensis miller (aloe vera), Morinda citrifolia (noni), and Boesenbergia rotunda (finger root).

Materials and methods

A total of 84 PMMA samples were used and were divided into three groups. Flexural strength tests were performed on Group 1 PMMA blocks. Group 2 involved hardness testing of PMMA blocks, whereas Group 3 involved antimicrobial activity. Each group was subsequently split into seven subgroups with differing concentrations of AgNPs: Sub Group 1: control (no AgNPs), Sub Group 2: 0.05% aloe vera AgNPs, Sub Group 3: 0.1% aloe vera AgNPs, Sub Group 4: 0.05% noni AgNPs, Sub Group 5: 0.1% of noni AgNPs, Sub Group 6: 0.05% finger root AgNPs, and Sub Group 7: 0.1% finger root AgNPs. The flexural strength was evaluated using a universal testing machine (Instron 8801). Surface hardness was measured using a Vickers tester (Tukon 1102). For the antimicrobial activity analysis, the samples were incubated in a suitable culture broth containing Candida albicans for 24 hours. Microbial colony count (colony-forming unit (CFU)/mL) was estimated to evaluate the microbial adhesion to the surface of the denture base materials.

Statistical analysis

The flexural strength, hardness, and CFU between the groups were analyzed using one-way analysis of variance (ANOVA) followed by multiple comparisons with Tukey’s honest significant difference (HSD) test (α=0.05). The level of statistical significance was determined at p<0.05.

Results

It was observed that the mean flexural strength was maximum in PMMA incorporated with 0.05% of aloe vera AgNPs and least in PMMA incorporated with 0.1% noni AgNPs. It was seen that a steady loss in flexural strength is observed from 0.05% to 0.1%. The mean hardness was maximum in PMMA incorporated with 0.1% of noni AgNPs and least in PMMA incorporated with 0.05% aloe vera AgNPs. It was also found that the hardness was directly proportional to the number of nanoparticles. With an increase in the weight percentage of nanoparticles, a steady increase in hardness was seen in all the test groups.

In our study, the results showed that finger root 0.1% showed the least CFU with a significant reduction of C. albicans adherence; therefore, it indicates higher anti-fungal activity. Aloe vera 0.05% showed the lowest inhibition of C. albicans, suggesting the least anti-fungal activity.

Conclusion

Within the limitations of this study, It can thus be concluded that the addition of AgNPs incorporated with plant extracts of Aloe barbadensis miller (aloe vera), Morinda citrifolia (noni), and Boesenbergia rotunda (finger root) can alter the flexural strength, hardness, and microbial adhesion of PMMA. In our study, it can be concluded that flexural strength increases with the addition of AgNPs of 0.5% concentration after which a steady loss is seen. However, the hardness and antimicrobial activity increased with an increase in the concentration of AgNPs in all three plant extracts.

## Introduction

Polymethylmethacrylate (PMMA) has been used widely as a denture base resin for many decades. However, PMMA was first used as a denture base resin for dentures in the 1930s. The drawbacks of denture base resin include its porosity, irregularity, and absorption, which can cause an accumulation of microorganisms that leads to the growth and formation of biofilms on the dentures. Over the years, fillers of different shapes, sizes, forms, and orientations have been added to denture base resins to alter mechanical properties. With the advent of nanotechnology, nanofillers are increasingly being used to improve the mechanical properties of denture base resin [1]. Nanoparticles (NPs) are preferred due to their superior dispersion properties, less aggregation potential, and biocompatibility with organic polymers and have found their application in various biomedical fields [2]. Silver nanoparticles (AgNPs) have been incorporated to enhance their antimicrobial properties and prevent biofilm accumulation. Their extremely small droplet size, high kinetic stability, low viscosity, and optical transparency have made them a common agent in the pharmaceutical industry as well [3]. AgNPs have also been shown to affect the mechanical properties of denture base resin such as hardness and flexural strength. The addition of nano-sized particles is characterized by better processing, smoother surfaces, and a large total surface area. This led to the introduction of AgNPs in denture base resin to provide superior properties to the dentures [4]. Biological methods using plant extracts make the NPs more biocompatible, environmentally benign, and cost-effective. Several biologically active constituents can be combined with AgNPs to yield non-toxic and chemically inert AgNP for denture base resins [5]. Aloe barbadensis miller (aloe vera) leaves have been used as medicinal plants since they possess anti-inflammatory and antibacterial activity [6-8]. Also, Boesenbergia rotunda (finger root) and Morinda citrofolia commonly called noni can function to prevent Candida albicans growth and oral infections from forming biofilms [2,5,9].

In this study, we have used Aloe barbadensis miller (aloe vera), Morinda citrofolia (noni), and Boesenbergia rotunda (finger root) extracts incorporated with AgNPs to evaluate the anti-fungal activity, flexural strength, and hardness of PMMA specimens.

## Materials and methods

This in vitro study was conducted at SRM Kattankulathur Dental College and Hospital, Chennai, India. The study was approved by the Institutional Scientific and Ethical Review Board of SRM Kattankulathur Dental College and Hospital before the commencement of the study (approval number: 2326/IEC/2020). The sample size was estimated using G*Power 3.1.9.2 software with 90% power.

Selection and collection of plant material

Three different natural plants were selected for the AgNPs synthesis. The powders of Aloe barbadensis miller (aloe vera), Morinda citrofolia (noni), and Boesenbergia rotunda (finger root) were collected. The powders were washed with de-ionized water by heating the de-ionized water at 70° on a hot plate stirrer. The solution was then filtered using a Whatmann No. 1 filter paper [7].

Synthesis of silver nanoparticles

Silver nitrate powder (AgNO_3_, Sigma-Aldrich) was added to the solution after the extract had been filtered to produce AgNPs. At room temperature, distilled water was used to adjust the AgNO_3_ powder concentration to 0.001 M, and the final volume was adjusted to 50 mL by adding the appropriate amount of de-ionized water. The solution was heated at a temperature of 70°C for six hours. For AgNPs, the solution turned from light brown to dark brown. The AgNPs were isolated and purified by centrifugation and then redispersed with the plant extract. AgNP powder was dehydrated for 12 hours at 80°C in a hot air oven. The NPs were then manually ground in an agate mortar and pestle to create AgNP powder. AgNP powder was mixed into the commercial heat-cured acrylic resin PMMA powder (DPI Heat Cure, DPI, Mumbai, Maharashtra, India) with various AgNP powder loadings (i.e., 0.05, 0.1wt%) [2,10].

Fabrication of specimens

A wax pattern of 3.3 mm thickness, 64 mm long, and 10 mm width was fabricated using modeling wax. The Type II model plaster was used to invest in the wax pattern. A second pour was done after applying a cold mold seal. Gypsum was allowed to completely dry before the wax removal process began. To accomplish wax removal, the denture flask is immersed in boiling water for 4 minutes. The flask is then removed from the water, and the appropriate segments are separated. Cold mold seal is applied onto the surfaces of the mold cavity. The packing was carried out using heat-cure PMMA powder (DPI Heat Cure, Dental Products of India, Mumbai, Maharashtra, India) combined with measured amounts of the prepared NPs at a concentration of 0.05% and 0.1%. The mixture was added with MMA monomer until the dough stage. After that, the flask is subjected to a hydraulic press to eliminate extra flash. After progressively raising the water bath's temperature to 100°C, the processing was done for one hour [11]. Following the curing cycle, the acrylic samples were removed and bench-cooled for 30 minutes. Using acrylic trimmers and sandpaper, the acrylic samples were examined for any discrepancies [11]. Using the same method, PMMA discs of 10 mm diameter and 2 mm thickness were fabricated to assess the antimicrobial activity. In our current study, a total of 84 samples were taken. A total of 28 samples (Group 1) were PMMA blocks, which were tested for flexural strength; 28 samples (Group 2) were PMMA blocks, which were tested for hardness; 28 samples (Group 3) were PMMA discs, which were tested for antimicrobial activity. Each group was further divided into seven subgroups (n=4): control (without AgNPs), 0.05% aloe vera AgNPs, 0.1% aloe vera AgNPs, 0.05% noni AgNPs, 0.1% noni AgNPs, 0.05% finger root AgNPs, and 0.1% finger root AgNPs.

Evaluation of flexural strength

**Figure 1:**
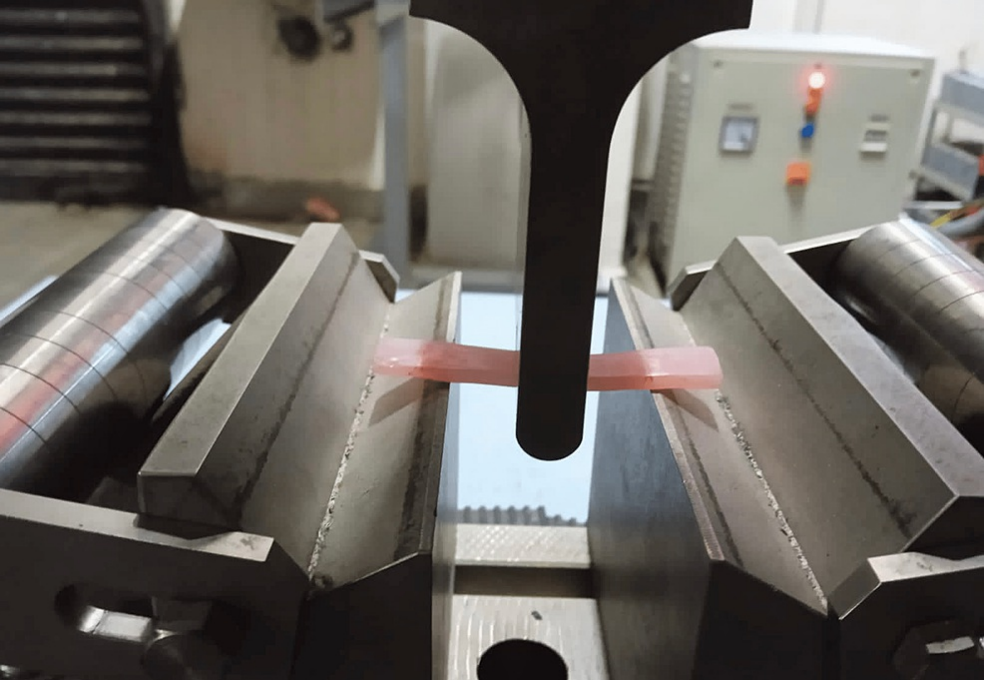
Evaluation of flexural strength The flexural strength of the samples was analyzed using a two-point bending test in a universal testing machine.

A three-point bending test was used to determine the flexural strength using a computerized universal testing apparatus (Instron 8801, United Kingdom) with a cross-head speed of 2 mm/minute [12] (Figure 1).

Evaluation of hardness

**Figure 2:**
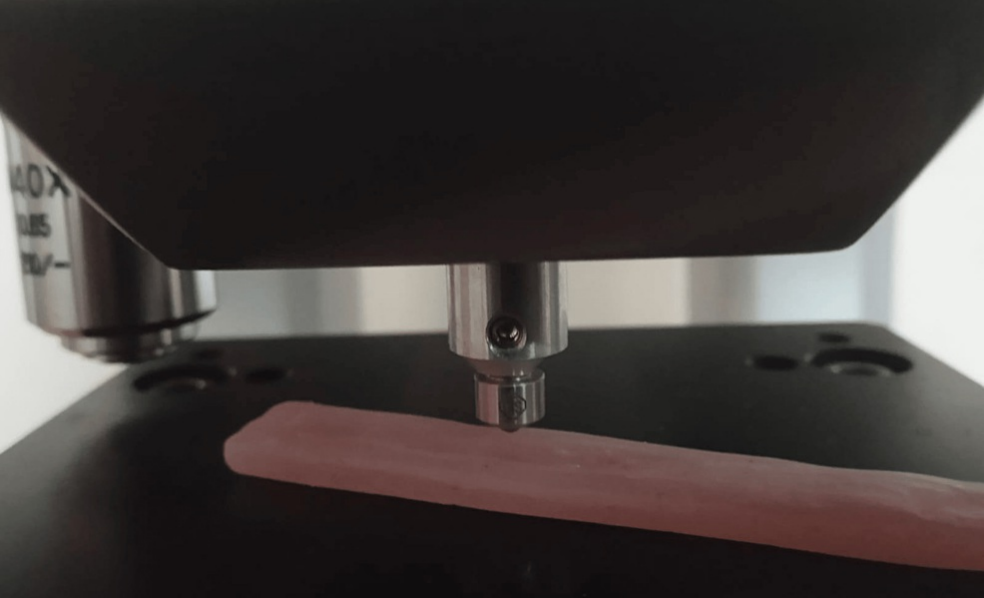
Evaluation of hardness The surface roughness of the samples was evaluated using a diamond pyramid-shaped indenter.

Vickers tester was used to determine surface hardness (Tukon 1102). On the surface of acrylic specimens, a 300 g load was applied perpendicularly for 15 seconds using a diamond pyramid-shaped indenter [13] (Figure 2).

Evaluation of antimicrobial effect (anti-fungal effect)

**Figure 3:**
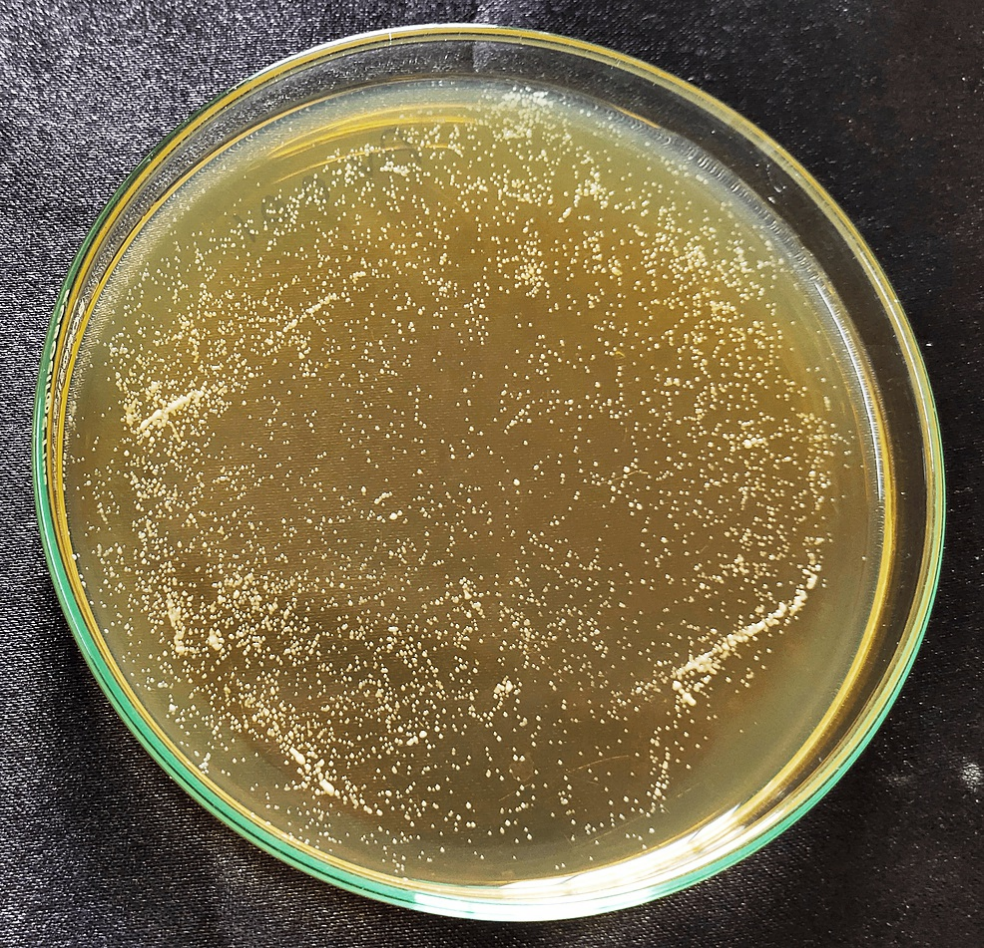
Control disc Cell count after Candida fungal suspension was spread onto a YM agar plate and incubated at 37°C for the control group (PMMA) without nanoparticles. PMMA, polymethylmethacrylate; YM, yeast malt

**Figure 4:**
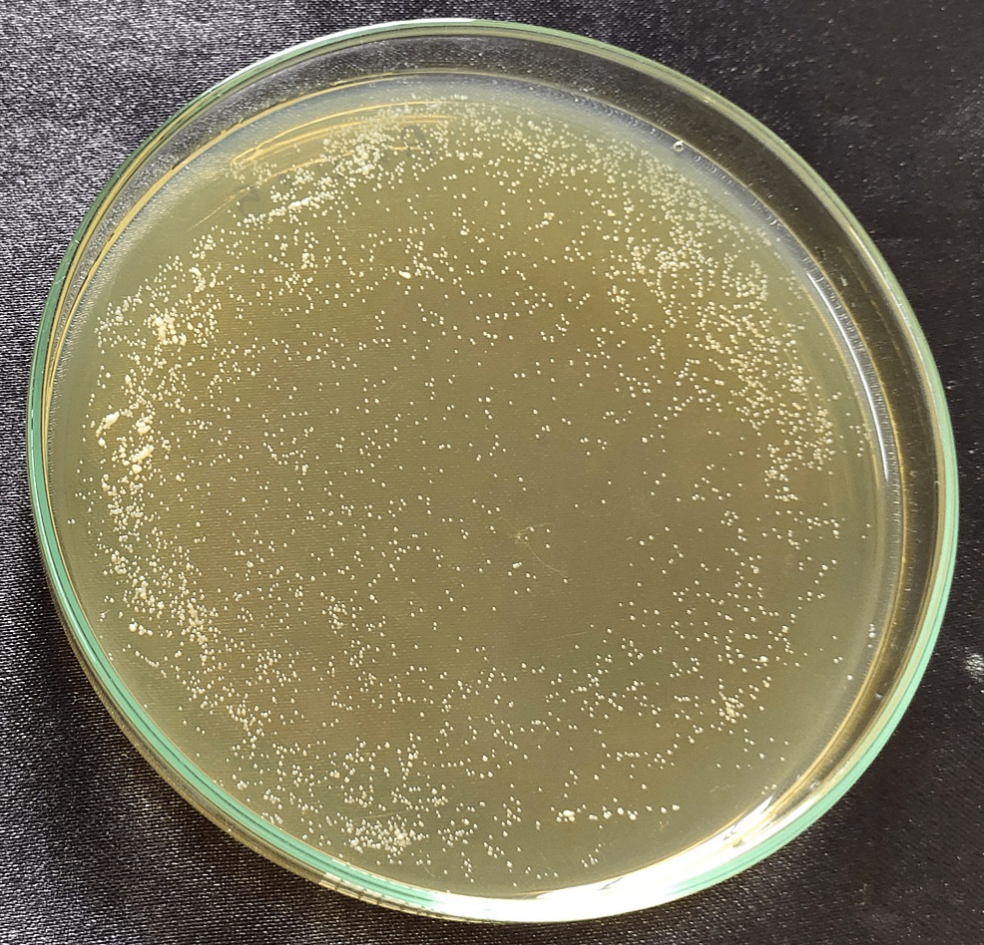
: Aloe vera 0.05% Cell count after Candida fungal suspension was spread onto YM agar plate and incubated at 37°C for the group: 0.05% aloe vera nanoparticles. YM, yeast malt

**Figure 5:**
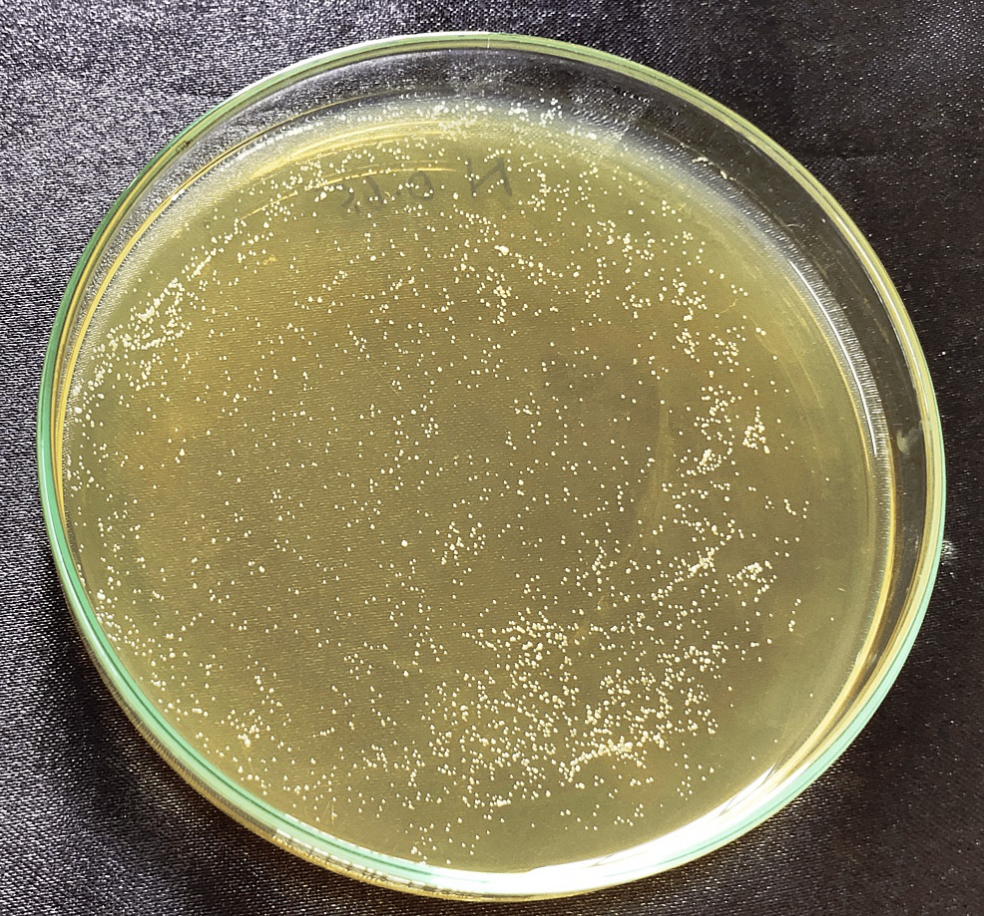
Noni 0.05% Cell count after Candida fungal suspension was spread onto YM agar plate and incubated at 37°C for the group: 0.05% noni nanoparticles. YM, yeast malt

**Figure 6:**
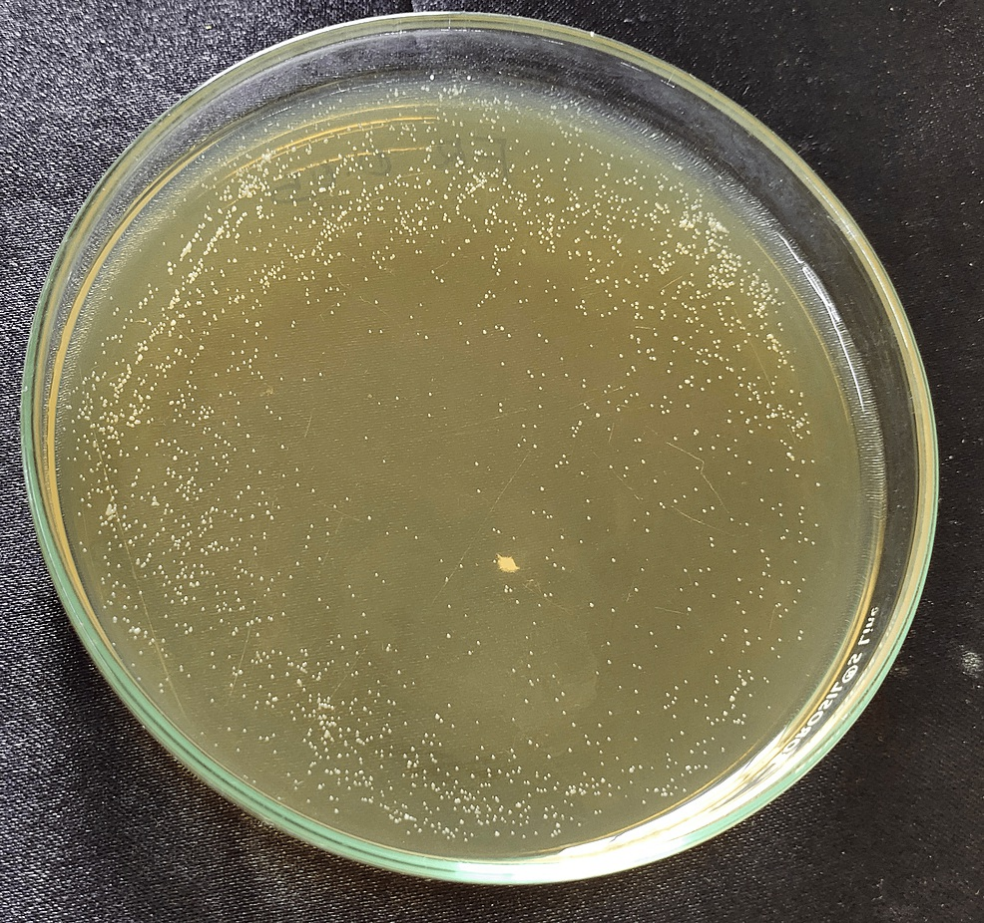
Finger root 0.05% Cell count after Candida fungal suspension was spread onto YM agar plate and incubated at 37°C for the group: 0.05% finger root nanoparticles. YM, yeast malt

**Figure 7:**
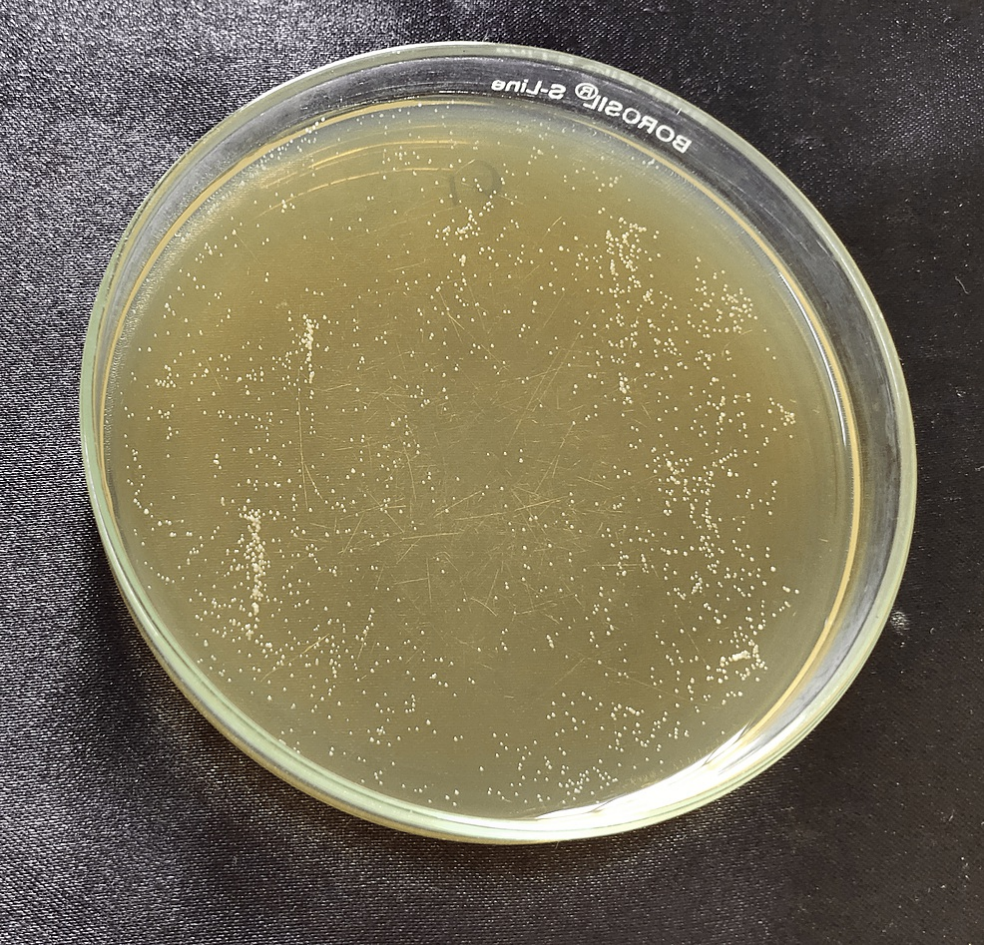
: Aloe vera 0.1% Cell count after Candida fungal suspension was spread onto YM agar plate and incubated at 37°C for the group: 0.1% aloe vera nanoparticles. YM, yeast malt

**Figure 8:**
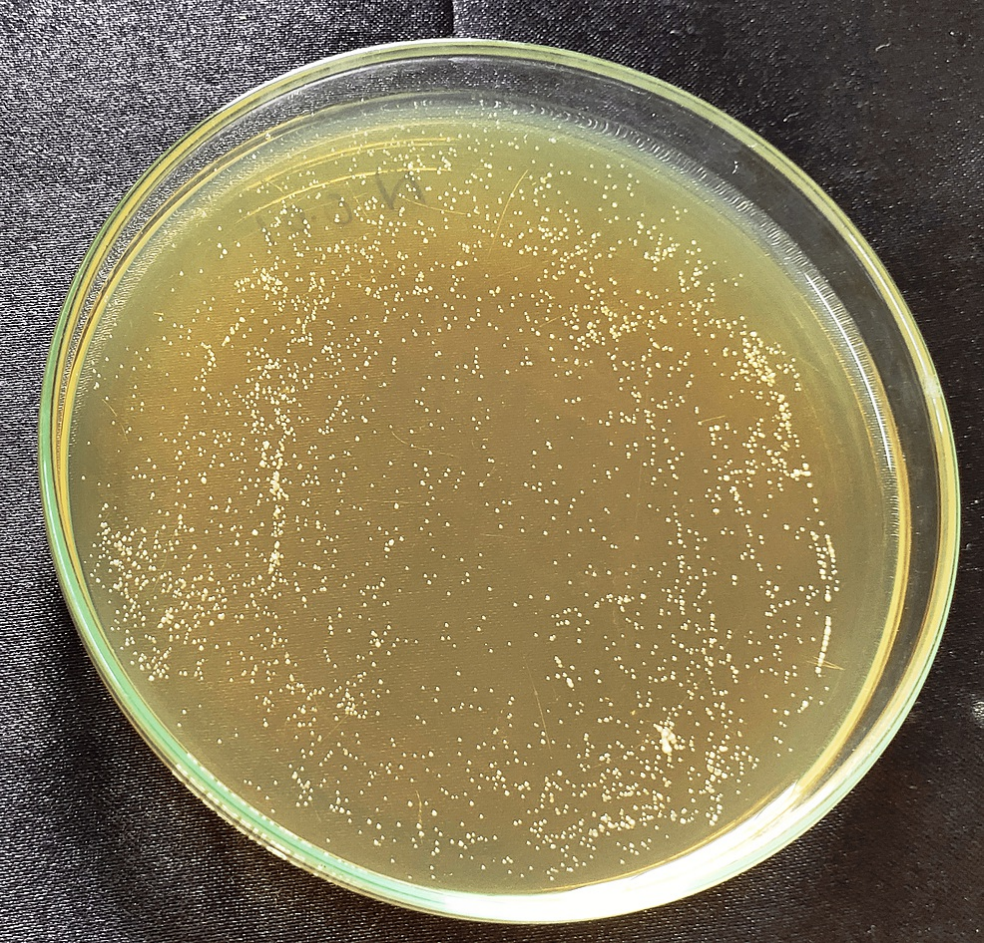
Noni 0.1% Cell count after Candida fungal suspension was spread onto YM agar plate and incubated at 37°C for the group: 0.1% noni nanoparticles. YM, yeast malt

**Figure 9:**
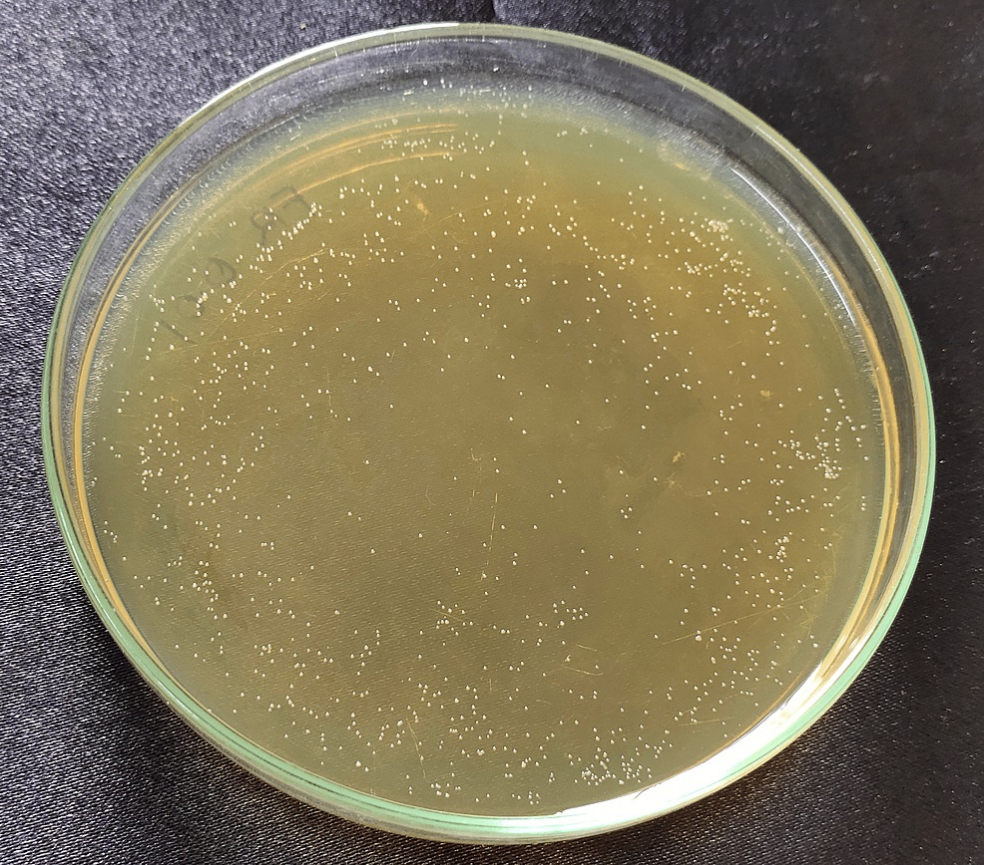
Finger root 0.1% Cell count after Candida fungal suspension was spread onto yeast malt agar plate and incubated at 37°C for the group: 0.1% finger root nanoparticles. YM, yeast malt

The strain used in the experiment was C. albicans (ATCC 10231). After preparing the disk-shaped specimens, 1 mL of fungal suspension (1×108 cells/mL) was placed onto each disk and incubated at 37°C for 24 hours. To evaluate the fungal colony-forming unit (CFU), the adherent fungi were harvested in 1 mL of YM (yeast malt) by sonication (SH-2100; Saehan Ultrasonic, Seoul, Korea) for 5 minutes. 100 μL of this fungal suspension was spread onto a YM agar plate and incubated at 37°C for 24 hours; the cells were counted and transformed to cells/ml. The cell count data were analyzed by one-way analysis of variance (ANOVA) followed by Tukey’s honest significant difference (HSD) test [14] (Figures 3-9).

## Results

The flexural strength, hardness, and CFU between the groups were analyzed using ANOVA followed by multiple comparisons with Tukey’s HSD test (α=0.05). The level of statistical significance was determined at p<0.05.

Based on the intergroup comparative analysis (ANOVA), it was found that PMMA denture material containing 0.05% of aloe vera NPs showed the highest flexural strength, and PMMA denture material containing 0.1% of noni AgNPs showed the least flexural strength. PMMA denture material containing 0.1% of noni AgNPs showed the highest hardness and PMMA containing 0.05% of aloe vera AgNPs showed the least hardness. It was also seen that PMMA denture material containing 0.1% finger root AgNPs significantly decreased the number of cultivable cells in the C. albicans biofilm compared to control by showing the least CFU whereas aloe vera 0.05% showed the highest CFU (Table 1, Table 2, and Table 3).

**Table 1:** Intergroup comparison of flexural strength between control and experimental groups

Flexural Strength (N/mm)	n	Mean + SD	Std. Error	95% Confidence Interval for Mean		Minimum	Maximum
				Lower Bound	Upper Bound		
Control	4	107.64 + 2.07	1.03827	104.3403	110.9487	104.83	109.78
Aloe Vera 0.05%	4	129.71 + 16.35	8.17676	103.6956	155.7399	107.02	143.96
Aloe Vera 0.1%	4	81.80 + 12.42	6.21256	62.0361	101.5785	69.07	98.88
Fin Root 0.05%	4	120.48 + 13.86	6.93493	98.4140	142.5540	107.55	137.40
Fin Root 0.1%	4	106.04 + 9.28	4.64173	91.2752	120.8193	98.34	118.01
Noni 0.05%	4	114.89 + 7.17	3.58589	103.4845	126.3082	108.72	124.90
Noni 0.1%	4	45.10 + 9.05	4.52571	30.7017	59.5073	35.70	56.78
Total	28	100.81 + 28.77	5.43749	89.6577	111.9713	35.70	143.96

**Table 2:** Intergroup comparison of hardness between control and experimental groups HV, Vickers hardness

Hardness (HV)	n	Mean + SD	Std. Error	95% Confidence Interval for Mean		Minimum	Maximum
				Lower Bound	Upper Bound		
Control	4	23.57 + 1.27	.63819	21.5440	25.6060	22.60	25.40
Aloe Vera 0.05%	4	21.65 + 0.60	.30139	20.6909	22.6091	20.80	22.20
Aloe Vera 0.1%	4	21.07 + 1.85	.92500	18.1312	24.0188	19.20	23.20
Fin Root 0.05%	4	30.87 + 1.12	.56476	29.0777	32.6723	29.80	32.40
Fin Root 0.1%	4	23.82 + 0.38	.19311	23.2104	24.4396	23.30	24.20
Noni 0.05%	4	24.82 + 0.69	.34731	23.7197	25.9303	24.10	25.60
Noni 0.1%	4	33.45 + 2.47	1.23996	29.5039	37.3961	30.00	35.40
Total	28	25.61 + 4.61	.87130	23.8230	27.3985	19.20	35.40

**Table 3:** Intergroup comparison of antimicrobial effect between control and experimental groups CFU, colony-forming unit

Antimicrobial effect (CFU)	n	Mean + SD	Std. Error	95% Confidence Interval for Mean		Minimum	Maximum
				Lower Bound	Upper Bound		
Control	4	456.00 + 22.92	11.46008	419.5289	492.4711	440.00	490.00
Aloe Vera 0.05%	4	425.00 + 27.64	13.82027	381.0177	468.9823	390.00	450.00
Aloe Vera 0.1%	4	215.75 + 13.12	6.56220	194.8661	236.6339	200.00	228.00
Fin Root 0.05%	4	258.50 + 22.29	11.14675	223.0261	293.9739	240.00	290.00
Fin Root 0.1%	4	188.50 + 8.69	4.34933	174.6585	202.3415	180.00	200.00
Noni 0.05%	4	256.25 + 31.98	15.99153	205.3578	307.1422	210.00	280.00
Noni 0.1%	4	255.00 + 22.73	11.36515	218.8310	291.1690	235.00	285.00
Total	28	293.57 + 100.03	18.90434	254.7829	332.3599	180.00	490.00

Based on the post hoc Tukey test of intergroup comparison, it was concluded that for the flexural strength, the group containing 0.1% noni AgNPs showed the highest significant difference in comparison with the other groups. For the hardness, denture base material containing 0.1% noni AgNPs showed the highest significant difference in comparison with the other groups. For the antimicrobial activity, the group containing 0.1% finger root AgNPs showed the highest significant difference in comparison with the other groups.

## Discussion

In our in vitro study, conventional PMMA denture base material was compared to denture base material incorporated with 0.05% and 0.1% AgNPs of Aloe barbadensis miller (aloe vera), Morinda citrifolia (noni) and Boesenbergia rotunda (finger root). Numerous studies have uncovered the effect of nanoparticle incorporation on the antimicrobial activity of polymers. Among the various NPs, AgNPs received particular interest from the researchers as they show a broad spectrum of antimicrobial activity. Also, it has been previously reported that AgNPs increase the surface hardness and that the hardness was directly proportional to the concentration of NPs incorporated in the denture base resin [10,15]. Additionally, it has been seen that NPs made using a biological process are more ecologically friendly and biocompatible. Additionally, the procedure is economical [2,4,7]. According to Grindlay and Reynolds, aloe vera has a variety of natural components including saponin, tannin, terpenoids, and flavonoids that can interact with microbial membranes. This contact with the cell membrane ultimately results in cell lysis [6]. It has been shown that environmentally benign AgNPs have been synthesized using Morinda citrifolia plant extracts. The synthesis is found to be efficient in terms of reaction time as well as the stability of the synthesized AgNPs [9]. Boesenbergia rotunda (finger root) can prevent oral infections by preventing the growth of C. albicans biofilms. Therefore, AgNPs can be produced with Boesenbergia rotunda plant extract, which can be used as the reducing agent that further increases the antimicrobial effect [2]. For the above reasons, in our study, AgNPs incorporated with plant extracts of Aloe barbadensis miller (aloe vera), Morinda citrofolia (noni), and Boesenbergia rotunda (finger root) have been used, which are frequently found in Asian countries.

Based on the statistical analysis, it was concluded that PMMA denture material containing 0.05% of aloe vera AgNPs showed the highest flexural strength and PMMA denture material containing noni 0.1% showed the least flexural strength. It has been seen that the mechanical properties of particulate-filled polymers are greatly influenced by the size, shape, and distribution of filler particles inside the polymer matrix [12,13]. The amount of particle dispersion inside the matrix has a significant impact on the strength of PMMA. Since the AgNPs incorporated in PMMA have a high surface area, the applied stress is predicted to transfer easily from the matrix to the AgNPs, improving their mechanical properties. Furthermore, there are stronger contacts that are created between the PMMA chains and AgNPs, which presumably enhance the mechanical strength and improve the compatibility between the polymeric matrix and the NPs. An important factor in determining how effectively a resin will function under the strain of mastication or chewing is the flexural strength of dentures. The flexural strength of acrylic resins can be affected positively or negatively by changes in the ratio of NPs [12].

In our in vitro study, we discovered that adding aloe vera, noni, and finger root AgNPs to the PMMA matrix at a concentration of 0.05% increased the material's flexural strength when compared to the control. But when it was measured at a concentration of 0.1%, it showed a lower value. It is seen that a steady loss in flexural strength is observed from 0.05% to 0.1%. This is in accordance with the study done by Alla R et al. which states that as the concentration of NPs in the polymer rises, it leads to a disordered structure of the PMMA, which reduces the contact between the metal nanoparticle and the polymer. This consequently leads to a decrease in mechanical strength. When present in greater quantities, NPs may also function as polymerization contaminants. As a result, the matrix may include a greater proportion of unreacted monomers, which would reduce its flexural strength [13].

For the hardness testing, PMMA denture material containing 0.1% of noni AgNPs showed the highest hardness and PMMA denture material containing 0.05% of aloe vera AgNPs showed the least hardness. Our results were as per previous literature by Alla RK et al. that showed that an increase in AgNP concentration increased Vickers hardness of denture base resins. They also added that if the correct proportions of AgNPs are employed, the incorporation of AgNPs may not have a negative impact on the mechanical properties of denture base resin with AgNPs [13].

For the antimicrobial testing, aloe vera 0.05% showed the lowest inhibition of C. albicans, suggesting the least antimicrobial activity. Manikandan et al. stated that protein denaturation and cell death are caused by the silver ions released by the AgNPs, which may adhere to the bacterial cell wall. AgNPs preferentially adhere to the cytoplasmic membrane, causing cell injury. Additionally, the AgNPs cause the death of bacteria or fungi by the pitting in the cell wall of bacteria or fungi [16]. Though there is widespread use of denture base materials in dentistry, there is limited literature comparing various plant extracts incorporated with AgNPs and their effect on flexural strength, hardness, and antimicrobial activity on denture base resins.

Limitations 

The limitation of the present in vitro study is that this study involved the use of only one common microorganism, whereas the oral cavity has different varieties of other microorganisms. It failed to assess the depth of penetration of microorganisms on the materials. The number of fungal colonies was evaluated only quantitatively. Another limitation could be that only one type of PMMA denture base resin was used. In addition to material limitations, the testing was performed in a lab setting that did not mimic the exact oral conditions, whereby the wet environment and simultaneous stresses were not simulated. Therefore, further in vivo investigations on the effect of different nanoparticle concentrations would be beneficial.

## Conclusions

The following conclusions can be made from our study. The mean flexural strength was maximum in PMMA incorporated with 0.05% of aloe vera AgNPs and least in PMMA incorporated with 0.1% noni AgNPs. The mean hardness was maximum in PMMA incorporated with 0.1% of noni AgNPs and least in PMMA incorporated with 0.05% aloe vera AgNPs. The mean CFU was least in PMMA incorporated with 0.1% finger root AgNPs indicating the highest antimicrobial activity. The mean CFU was maximum in PMMA incorporated with 0.05% of aloe vera AgNPs indicating the lowest antimicrobial activity. It is important to provide a prosthesis with high flexural strength and hardness. These physical properties play an important role in the longevity of long-term dentures. The tendency of a denture base material to adhere with the microorganisms should also be considered since these prostheses will be in direct contact with the microflora of the mouth and might lead to biofilm formation on the surfaces that can cause infections locally or systemically. Therefore, further in vivo studies should aim to search for new materials with superior physical properties and the least microbial adhesion.

Nonetheless, this current study reported the differences in flexural strength, hardness, and antimicrobial activity among denture base materials incorporated with different types of NPs, which is a significant merit to the study.

## References

[1] Aldegheishem A, AlDeeb M, Al-Ahdal K, Helmi M, Alsagob EI (2021). Influence of reinforcing agents on the mechanical properties of denture base resin: a systematic review. Polymers (Basel).

[2] Siripanth J, Wongwitthayakool P (2018). Flexural strength and viscoelastic properties of acrylic resin denture base material containing silver nanoparticle synthesized from the fingerroot aqueous extract. Key Eng Mater.

[3] Salih SI, Oleiwi JK, Mohamed AS (2018). Investigation of mechanical properties of PMMA composite reinforced with different types of natural powders. J Eng Appl Sci.

[4] Tippayawat P, Phromviyo N, Boueroy P, Chompoosor A (2016). Green synthesis of silver nanoparticles in aloe vera plant extract prepared by a hydrothermal method and their synergistic antibacterial activity. PeerJ.

[5] Abou Assi R, Darwis Y, Abdulbaqi IM, khan AA, Vuanghao L, Laghari MH (2017). Morinda citrifolia (Noni): a comprehensive review on its industrial uses, pharmacological activities, and clinical trials. Arab J Chem.

[6] Grindlay D, Reynolds T (1986). The Aloe vera phenomenon: a review of the properties and modern uses of the leaf parenchyma gel. J Ethnopharmacol.

[7] Burange PJ, Tawar MG, Ritu AB, Vedanshu RM (2021). Synthesis of silver nanoparticles by using Aloe vera and Thuja orientalis leaves extract and their biological activity: a comprehensive review. Bull Natl Res Cent.

[8] Qian Y, Yao J, Russel M, Chen K, Wang X (2015). Characterization of green synthesized nano-formulation (ZnO-A. vera) and their antibacterial activity against pathogens. Environ Toxicol Pharmacol.

[9] Asha Pai, S Kavitha, Shwetha Raj (2014). Green synthesis and characterization of silver nanoparticles using fresh leaf extracts of Morinda citrofolia and its antimicrobial activity studies. J Pharm Pharm Sci.

[10] Logeswari P, Silambarasan S, Abraham J (2015). Synthesis of silver nanoparticles using plants extract and analysis of their antimicrobial property. J Saudi Chem Soc.

[11] Sultana N, Ahmed S, Nandini VV, Lathief J, Boruah S (2023). An in vitro comparison of microbial adhesion on three different denture base materials and its relation to surface roughness. Cureus.

[12] Alla R, Swamy K, Vyas R, Konakanchi A, Guduri V (2017). Gadde pinfluence of silver nanoparticles incorporation on flexural strength of heat-cure acrylic denture base resin materials. Annu Res Rev Biol.

[13] Alla RK, Guduri V, Tiruveedula NBP, Rao GN, Swamy KNR, Vyas R (2020). Effect of silver nanoparticles incorporation on microhardness of Heat-cure denture base resins. Int J Dent Mater.

[14] Lee MJ, Kim MJ, Oh SH, Kwon JS (13). Novel dental poly (methyl methacrylate) containing phytoncide for antifungal effect and inhibition of oral multispecies biofilm. Int J Dent Mater.

[15] Alhotan A, Yates J, Zidan S, Haider J, Silikas N (2021). Flexural strength and hardness of filler-reinforced PMMA targeted for denture base application. Materials (Basel).

[16] Manikandan V, Velmurugan P, Park JH (2017). Green synthesis of silver oxide nanoparticles and its antibacterial activity against dental pathogens. 3 Biotech.

